# pH-Sensitive Fluorescent Probe in Nanogel Particles as Theragnostic Agent for Imaging and Elimination of Latent Bacterial Cells Residing Inside Macrophages

**DOI:** 10.3390/gels10090567

**Published:** 2024-08-30

**Authors:** Igor D. Zlotnikov, Alexander A. Ezhov, Natalya G. Belogurova, Elena V. Kudryashova

**Affiliations:** 1 Faculty of Chemistry, Lomonosov Moscow State University, Leninskie Gory, 1/3, 119991 Moscow, Russia; zlotnikovid@my.msu.ru (I.D.Z.);; 2 Faculty of Physics, Lomonosov Moscow State University, Leninskie Gory, 1/2, 119991 Moscow, Russia; alexander-ezhov@yandex.ru

**Keywords:** theranostics, nanogel particles, FRET marker, pH sensitivity, visualization of bacterial cells, intracellular macrophage infections

## Abstract

Rhodamine 6G (R6G) and 4-nitro-2,1,3-benzoxadiazole (NBD) linked through a spacer molecule spermidine (spd), R6G-spd-NBD, produces a fluorescent probe with pH-sensitive FRET (Förster (fluorescence) resonance energy transfer) effect that can be useful in a variety of diagnostic applications. Specifically, cancer cells can be spotted due to a local decrease in pH (Warburg effect). In this research, we applied this approach to intracellular infectious diseases—namely, leishmaniasis, brucellosis, and tuberculosis, difficult to treat because of their localization inside macrophages. R6G-spd-NBD offers an opportunity to detect such bacteria and potentially deliver therapeutic targets to treat them. The nanogel formulation of the R6G-spd-NBD probe (nanoparticles based on chitosan or heparin grafted with lipoic acid residues, Chit-LA and Hep-LA) was obtained to improve the pH sensitivity in the desired pH range (5.5–7.5), providing selective visualization and targeting of bacterial cells, thereby enhancing the capabilities of CLSM (confocal laser scanning microscopy) imaging. According to AFM (atomic force microscopy) data, nanogel particles containing R6G-spd-NBD of compact structure and spherical shape are formed, with a diameter of 70–100 nm. The nanogel formulation of the R6G-spd-NBD further improves absorption and penetration into bacteria, including those located inside macrophages. Due to the negative charge of the bacteria surface, the absorption of positively charged R6G-spd-NBD, and even more so in the chitosan derivatives’ nanogel particles, is pronounced. Additionally, with a pH-sensitive R6G-spd-NBD fluorescent probe, the macrophages’ lysosomes can be easily distinguished due to their acidic pH environment. CLSM was used to visualize samples of macrophage cells containing absorbed bacteria. The created nanoparticles showed a significant selectivity to model *E. coli* vs. *Lactobacillus* bacterial cells, and the R6G-spd-NBD agent, being a mild bactericide, cleared over 50% *E.coli* in conditions where *Lactobacillus* remained almost unaffected. Taken together, our data indicate that R6G-spd-NBD, as well as similar compounds, can have value not only for diagnostic, but also for theranostic applications.

## 1. Introduction

Nanomedicine and theranostics approaches in the development of pharmaceutical formulations that serve as both diagnostic tools and improved therapeutic agents is particularly relevant for the treatment of severe macrophage-associated diseases, including infectious, chronic inflammatory, and autoimmune diseases [1,2,3,4,5,6,7]. Intracellular infections, such as tuberculosis [8,9,10,11], chlamydia [12,13,14], brucellosis [15,16,17,18,19], and leishmaniasis [20,21,22], are difficult to treat largely due to their intracellular localization within macrophages. The mechanisms of the resistance, as well as the specific features of their interaction with macrophages, require further investigation in order to develop safer and more effective treatment regimens. Visualization of pathogenic bacteria within macrophages is one way to advance research in this area. However, despite advances in confocal laser scanning microscopy (CLSM) imaging of intracellular structures, this remains a significant challenge.

Tissue macrophages play a crucial role in maintaining tissue homeostasis and responding to injury and infection. They engage in highly coordinated and complex activities, including clearing dying cells, bacteria, and cellular debris. Classically activated macrophages, known as M1 macrophages, protect the body from microorganisms by initiating an inflammatory response through the release of pro-inflammatory cytokines [23,24,25,26,27,28,29]. Alternatively, activated (M2) macrophages help to regulate the response to injury and inflammation by producing anti-inflammatory cytokines [30,31,32,33,34,35]. Activated macrophages are known to absorb and ingest various nanoparticles, which, we assumed, could be a good starting point to design a fluorescent probe for infections residing inside macrophages.

Nanoparticles based on biopolymers offer several advantages, including improved drug distribution and accumulation and the ability to image pathogenic cells and areas of inflammation [36,37,38] where the nanoparticles can accumulate.

Biocompatible and biodegradable, polysaccharide nanogel particles have several undeniable advantages in terms of therapeutic efficacy and safety: (1) a specific size (50–200 nm) that allows them to remain in the bloodstream and penetrate into pathogenic cells (bacteria, parasite microorganisms, or tumors) using the features of their biomembrane structure [39]; (2) the enhanced permeability and retention (EPR) effect that promotes accumulation of nanoparticles in the area of inflammation or in the tumor microenvironment [40,41,42]; (3) biocompatible polymer carriers, such as chitosan or heparin, lead to improved targeting and safety, and reduce systemic toxicity [43]; and (4) the possibility of creating a combined drug for enhancing the efficiency of nanogel particles due to synergies of the drugs’ action, as we have recently shown in the works [44,45].

The targeted theranostics approach can be harnessed to deliver diverse pharmaceutical agents, encompassing nucleic acids, chemotherapeutic agents, and hyperthermal agents, among others. These substances can then be employed for a variety of subsequent applications, such as photothermal ablation, photodynamic therapy, and radiation treatment [5].

The application of theranostics has significant potential to shed light on the role of macrophages in regulating inflammation and developing strategies to treat chronic inflammation in serious infectious diseases, where intramacrophage infections may be “dormant” or latent, making them difficult to treat [46,47,48]. The example of a disease that requires a more efficient approach is intracellular macrophage-associated brucellosis infection—a severe disease, characterized by a variety of pathological changes in multiple organs and a propensity for chronicity [49,50,51,52,53]. Brucellosis is a highly virulent disease that exhibits severe symptoms, and its intracellular nature poses significant challenges for diagnosis and treatment. Therefore, exploring new approaches, such as theranostic nanogel formulations, is crucial in these cases.

In this paper, we propose a targeted drug delivery system of polysaccharide nanogel particles to diagnose pathogenic bacterial infections and, simultaneously, to treat these pathogens (and latent forms within macrophages). Thus, a fluorescent diagnostic agent and an antibiotic combined in a single biocompatible nanogel formulation is obtained.

The aim of this study is to develop a pH-sensitive fluorescent marker that could specifically stain bacterial cells, compared to normal cells, due to differences in their microenvironment. For this purpose, for the pH probe we propose a conjugated form of rhodamine 6G (R6G) and 4-nitro-2,1,3-benzoxadiazole (NBD), linked via a spacer molecule, spermidine (spd), R6G-spd-NBD, which, due to protonation, ensures the pH sensitivity of the fluorescent conjugate.

R6G exhibits maximum excitation at a wavelength of 525 nm, with a quantum efficiency of 0.95. NBD-spd, on the contrary, has maximum excitation at 488 nm and maximum emission at 545 nm. In the R6G-spd-NBD conjugated system, the fluorescence excitation spectrum is shifted towards shorter wavelengths relative to free R6G. This leads to a broader fluorescence emission peak due to Förster resonance energy transfer (FRET), with an efficiency of approximately 38%. The fixed interplanar distance between R6G and NBD is 8.7 Angstroms (Å), as was calculated earlier [1]. NBD-spd acts as an ideal fluorophore donor for R6G. Additionally, the enhancing effect of FRET is observed between the NBD donor and the R6G acceptor when the probe enters the target cells. The nanoparticles of chitosan- (or heparin)-lipoic acid (Chit-LA or Hep-LA) was applied to enhance the pH sensitivity to changes in acidity of the surrounding medium [39,54,55].

Therefore, we propose a polysaccharide-based nanogel formulation of R6G-spd-NBD for selective staining of bacterial cells in macrophages for the diagnosis and treatment of the intracellular infectious diseases leishmaniasis, brucellosis, and tuberculosis, which form latent particles inside macrophages. The key objectives are (i) R6G-spd-NBD synthesis and the creation and characterization of a nanogel formulation; (ii) investigation of the fluorescent properties of R6G-spd-NBD both in free and nanogel form, in solution and in vitro after exposure to E. coli or Lactobacillus cells; (iii) determination of the antibacterial effects of R6G-spd-NBD compared to R6G, and the correlation between these effects and permeability into bacterial cells; (iv) visualization of the penetration of R6G-spd-NBD into bacterial cells using confocal fluorescence microscopy; and (v) the study of model latent bacterial infections within macrophages, focusing on diagnosis and treatment and the discussion of the potential diagnostic and therapeutic applications of the products developed.

## 2. Results and Discussion

### 2.1. Work Design

**Figure 1:**
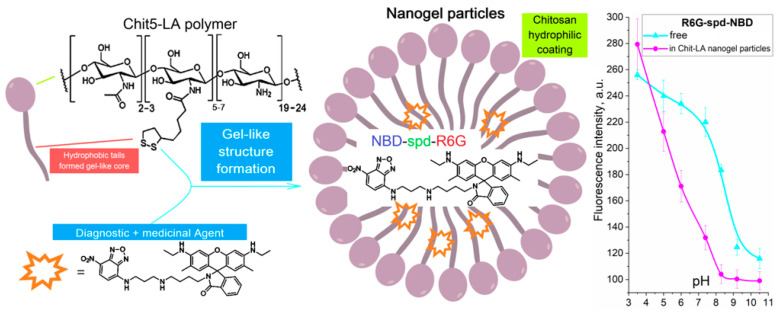
A schematic representation of the polysaccharide nanogel particles loaded with theranostic agent R6G-spd-NBD with a pH-sensitivity function. Left panel delineates the architectures of amphiphilic polymers and the fluorescent probe, the central panel presents a schematic depiction of nanogel particles, and the right panel illustrates the correlation between the fluorescence intensity at a wavelength of 550 nm and pH for a therapeutic formulation based on R6G-spd-NBD. The ellipsoidal shape is a schematic representation of the polar head of a polymer forming a gel nanoparticle composed of polymer chains derived from chitosan or heparin. The lines depict the hydrophobic tails of the acidic residues.

The aim of this study is to develop a theranostic agent R6G-spd-NBD, with regard to its antibacterial activity and its potential for pathogen diagnostics (Figure 1). The nanogel formulations of the R6G-spd-NBD probe, which is chitosan or heparin that has been grafted with lipoic acid residues (Chit-LA and Hep-LA, respectively), has been designed to improve sensitivity to changes in the intracellular environment and serves as a marker to distinguish between model bacterial cells of *Escherichia coli* (*E. coli*) and *Lactobacillus* species.

Figure 1 illustrates a schematic representation of the nanogel formulation with the theranostic agent R6G-spd-NBD. We used the amphiphilic polymer chitosan-lipoic acid (Chit-LA), as it spontaneously forms nanoparticles that, on the one hand, facilitate solubilization and show high loading efficiency (up to 85%) of the fluorophore, and on the other hand, allow for the selective release of the agent when exposed to a triggering stimulus (pH, temperature, or chemical stimuli). This would significantly enhance the diagnostic and therapeutic capabilities of R6G-spd-NBD. We used chitosan-lipoic acid nanoparticles (Chit-LA) with a positive charge (ζ = +8 mV), compared to heparin-lipoic acid particles (Hep-LA) with a negative charge (ζ = –12 mV).

### 2.2. Synthesis, Characterization, and Preparation of R6G-spd-NBD and Its Nanogel Formulation

**Figure 2:**
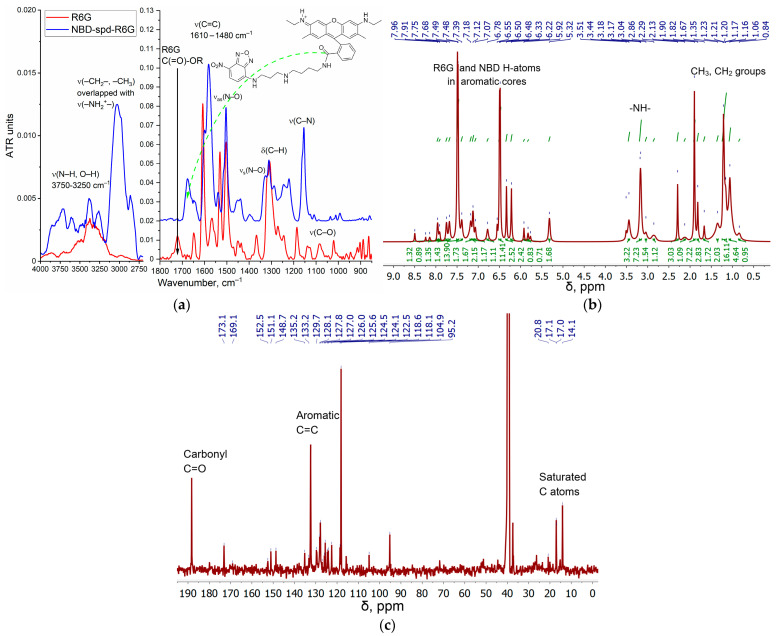
(**a**) FTIR spectra and structure of R6G-spd-NBD. PBS (0.01 M, pH = 7.4). (**b**) The ^1^H NMR spectra of R6G-spd-NBD. (**c**) The ^13^C NMR spectra of R6G-spd-NBD. T = 22 °C. DMSO-d_6_. Working frequencies: 500.13 MHz (^1^H) and 125.76 MHz (^13^C).

Figure 2a illustrates the conjugate structure of rhodamine 6G (R6G) and 4-nitro-2,1,3-benzoxadiazole (NBD), which are linked via a spacer molecule, spermidine (spd), as well as FTIR spectra of the R6G-spd-NBD fluorescent probe in comparison to R6G alone. The synthesis of R6G-spd-NBD was performed in 4 stages (Figure S1): (1) protection of spd NH_2_ group with 4-hydroxybenzaldehyde HO-C_6_H_4_-CHO by the formation of a Schiff base HO-C_6_H_4_-HC=N-spd, which was corroborated by the emergence of a pronounced FTIR peak at a wavelength of 1645 cm^−1^ ν(C=N) (Figure S2); (2) HO-C_6_H_4_-HC=N-spd was reacted with NBD-Cl via the S_N_Ar mechanism, with the formation of HO-C_6_H_4_-HC=N-spd-NBD; (3) deprotection of the spermidine NH₂ group resulted in the formation of NBD-spd, which was reflected in the increased intensity of the ν(N-H) peaks in the FTIR spectrum, specifically in the range from 3600 to 3300 cm^−1^ and from 3100 to 2750 cm^−1^, and disappearance of the ν(C=C) peaks of the 4-hydroxybenzaldehyde (1560–1615 cm^−1^) (Figure S2); and (4) the final stage involved the covalent bonding of NBD-spd with rhodamine 6G (R6G), resulting in the formation of an amide linkage between an ester group and an amine group, which was confirmed by observing certain spectral features: disappearance of the FTIR ν(ester C=O) peak in free R6G (1730–1710 cm^−1^) and the appearance of the FTIR peaks of ν(amide C=O) 1690–1650 cm^−1^ and δ(amide N–H) 1580 cm^−1^ (Figure 2a). FIIR spectroscopy confirmed the formation of the chemical conjugate R6G-spd-NBD, with additional validation of the drug being carried out using the complementary NMR spectroscopy method.

The following important signals are observed in the NMR proton spectrum of the substance NBD-spd (Figure S3): aromatic 7.8, 7.5 ppm (–CH=C(NO_2_)–) and 7.1, 6.9 ppm (–CH=C(NR)–), and signals referenced to the spermidine spacer in the range of 1.1–3.0 ppm, which are in a good agreement with the literature data for NBD and similar compounds [56,57,58,59]. Figure 2b,c shows the ^1^H and ^13^C NMR spectra of R6G-spd-NBD. In the ^1^H NMR spectrum of R6G-spd-NBD, signals are observed in the region of 6–8 ppm, which corresponds to protons in the aromatic system of R6G. In the ^13^C NMR spectrum, signals in the range 118–153 ppm are referenced to C-atoms in the aromatic systems of R6G or NBD. The ^13^C NMR signals at 14–21 ppm correspond to the C-atoms in CH_3_ and CH_2_ groups of spd spacer. The NMR peaks at 169 and 173 ppm correspond to the C-atoms in the amide bond between R6G and NBD-spd (Figure 2c). Therefore, NMR spectroscopy has confirmed the formation of R6G-spd-NBD.

Table 1 shows the main physico-chemical parameters of the synthesized polymers. Chitosan-lipoic acid nanoparticles (Chit-LA) have a positive charge (ζ = +8 mV), and in the contrary, heparin-lipoic acid particles (Hep-LA) have a negative charge (ζ = –12 mV). Non-loaded particles are approximately 210 ± 40 nm in size (hydrodynamic diameter). Chit-LA nanogel particles containing R6G-spd-NBD are approximately 100 ± 20 nm in size. Hep-LA nanogel particles empty and containing R6G-spd-NBD are approximately 310 ± 50 nm and 250 ± 70 nm in size, correspondingly.

**Figure 3:**
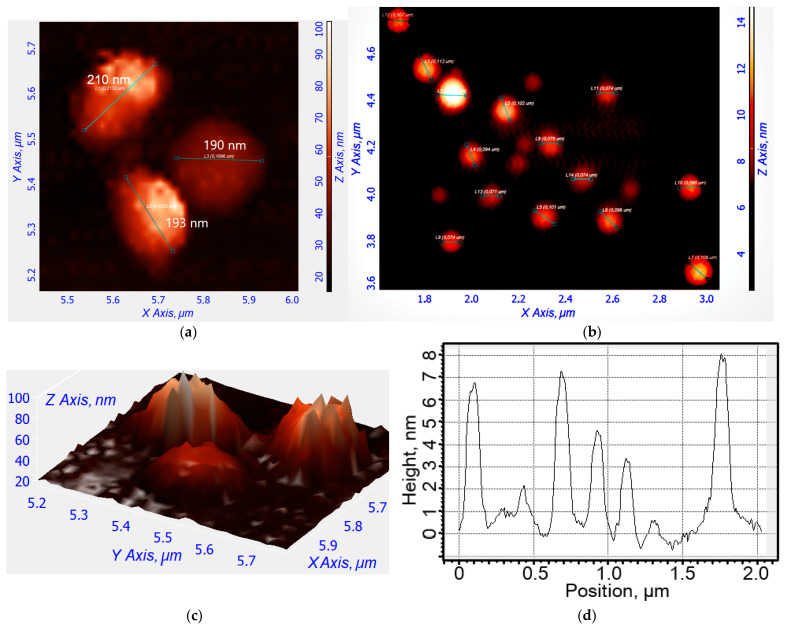
(**a**) Atomic force microscopy images of non-loaded Chit5-LA in nanoparticles in 2D view. (**b**) Atomic force microscopy images of Chit5-LA nanoparticles loaded with R6G-spd-NBD (10 mass.%). (**c**) Atomic force microscopy images of non-loaded Chit5-LA nanoparticles in 3D view. (**d**) The corresponding height section of (**b**) through the main diagonal from top to bottom.

The structure of the nanoparticles was investigated using atomic force microscopy (AFM), as shown in Figure 3. Empty particles have a distorted spherical shape and are approximately 190–210 nm in size. In this instance, the nanogel containing R6G-spd-NBD becomes compact (the aromatic molecules of the drug acting as a type of seed/primer) and take on a highly spherical shape with a diameter ranging from 70 to 110 nm. Nanogel particles undergo a process of compactization when loaded with a drug due to increased hydrophobic interaction in the presence of the aromatic molecules acting as the nuclei. The guest molecules serve as enhancers of the nanoparticles’ stability and promote their compactization.

### 2.3. Fluorescent Properties of the Theranostic Agent R6G-spd-NBD in the Polysaccharide Nanogel Particles and Interaction with Bacterial Cells

#### 2.3.1. Fluorescence Spectra

**Figure 4:**
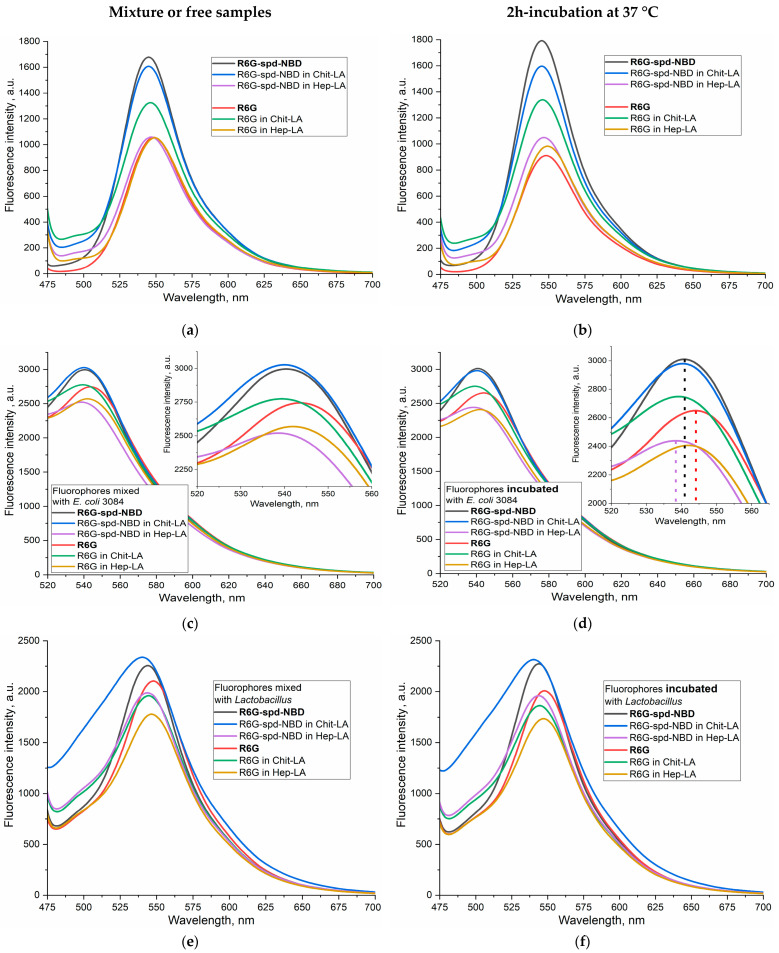
Fluorescence emission spectra of R6G (1 µM) and the pH sensor R6G-spd-NBD (1 µM): (1) in PBS buffer (**a**) prior to and (**b**) following a 2 h incubation at 37 °C; (2) mixed with an *E. coli* bacterial suspension (10^7^ CFU/mL) in PBS (**c**) prior to and (**d**) following a 2 h incubation at the same temperature; (3) mixed with a *lactobacillus* suspension (10^7^ CFU/mL) in PBS (**e**) prior to and (**f**) following the same incubation period at 37 °C. λ_exci_ = 460 nm. PBS (0.01 M, pH = 7.4). T = 37 °C.

Figure 4 shows the fluorescent spectra of the nanogel formulation of the R6G-spd-NBD compared with the control sample R6G in buffer solution (a,b), when exposed to *E. coli* (c,d), and to *Lactobacillus* bacteria (e,f). The spectra for mixtures (without incubation) and for samples after 2 h of incubation with cells at 37 °C were analyzed. We studied Chit-LA positively charged nanoparticles, in comparison to Hep-LA with a negative charge.

In the buffer solution, the fluorescence intensity of R6G-spd-NBD in the free form and in nanogel particles of Chit-LA is 60–70% higher than for R6G samples, while in the nanogel form of Hep-LA, the fluorescence intensity is almost the same for R6G-spd-NBD and R6G (Figure 4a,b). Thus, positively charged nanoparticles augment the fluorescence intensity of probes either slightly or not at all, whereas negatively charged particles diminish it. Specifically, for R6G-spd-NBD, the fluorescence intensity amounts to approximately 1680 units in its free form, 1600 in the presence of chitosan nanoparticles, and 1050 in the case of heparin-based particles.

When exposed to *E. coli* bacterial cells, an increase in the fluorescence intensity of both R6G-spd-NBD and R6G is observed by about 2 times, while in the case of *Lactobacilli*, ignition is only 40–50% (for both fluorophores), i.e., fluorophores are sensitive specifically to model pathogenic bacteria *E. coli* vs. non-pathogenic *lactobacilli*. When the fluorophore binds to bacterial cells, a shift to the short-wavelength region of the maximum fluorescence emission is observed (Figure 4).

R6G-spd-NBD, in its nanogel Chit-LA formulation, exhibits the highest fluorescence intensity associated with *E. coli* cells, making it the most suitable for use in the selective detection of pathogenic bacteria. Simultaneously, the sensitivity of the R6G-spd-NBD conjugate in its nanogel Chit-LA formulation exhibits a higher value compared to that of the formulation containing R6G, with an intensity of fluorescence measured at 3010 versus 2770 units, respectively.

To explain the observed effect, we turn to the pH dependence of R6G-spd-NBD in nanoparticles of Chit-LA, which demonstrated the pH sensitivity of the R6G-spd-NBD probe (Figure 1), also studied in detail in our recent work [54]. The data (Figure 1) indicate that the transition of the probe from the nanoparticles’ environment into different bacterial cells would affect its fluorescence intensity.

#### 2.3.2. The FRET Process in the Context of the Interaction between R6G-spd-NBD and Bacterial Cells

Besides pH sensitivity, the conjugate R6G-spd-NBD also provides the phenomenon of FRET. FRET occurs, when two fluorophores are within a distance of 1–10 nm of each other, within the overlapping emission and excitation spectra of two fluorophores. In the case of a NBD and R6G pair, the NBD exhibits a spectral maximum at 488 nm in terms of excitation, with its maximum emission occurring at 545 nm (Figure S4). On the other hand, R6G show a maximum excitation wavelength of 525 nm, and a maximum emission wavelength at 550 nm (Figure S4). When NBD-spd is conjugated with R6G to form R6G-spd-NBD, the fluorescence excitation shifts towards shorter wavelengths compared to free R6G, resulting in a broader emission peak due to the FRET effect. The efficiency of this process is approximately 38%, assuming a fixed interplanar distance between R6G and NBD of 8.7 angstroms [54]. As a result, NBD-spd serves as an efficient fluorophore donor for R6G in these conjugates.

**Figure 5:**
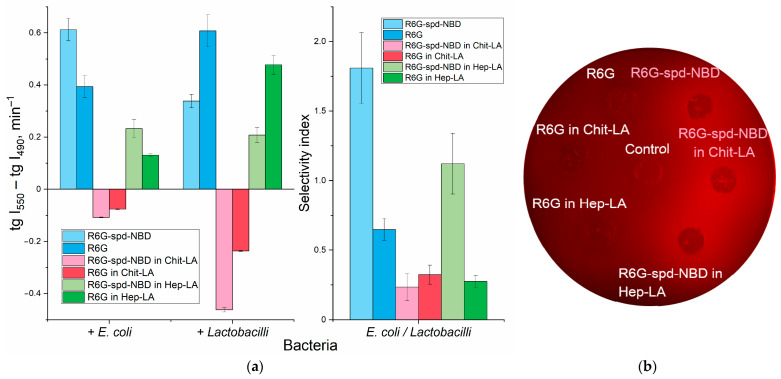
(**a**) Kinetic parameters for the 1 h interaction between R6G or R6G-spd-NBD pH sensors with *E. coli* and *lactobacillus* suspensions (10^7^ CFU/mL): the slopes of the fluorescence emission intensity curves at 490 nm (NBD > R6G) and 550 nm (R6G > NBD). The ratio of tangents of kinetic lines at 490 nm and 550 nm for *E. coli* and *lactobacillus*. λ_exci_ = 460 nm. PBS (0.01 M, pH = 7.4). T = 37 °C. (**b**) Fluorescent image of a Petri dish with *E. coli* cells (10^7^ CFU/0.5 mL placed onto 20 mL of agar medium) when exposed to R6G or R6G-spd-NBD in free form and in nanogel particles for 12 h at 37 °C. The fluorophores are placed in wells with a diameter of 9 mm. The concentration of fluorophores is 0.1 mg/mL, the concentration of amphiphilic polymers is 0.5 mg/mL. λ_exci, max_ = 480 nm. λ_emi_ = 515–570 nm.

An important parameter that characterizes the interaction between bacterial cells and fluorophores or nanogel formulations is the fluorescence intensity, and more specifically, its change. This parameter is measured as the derivative of the corresponding kinetic curves: Figure 5a shows the slope values for kinetic curves that characterize the interaction of R6G-spd-NBD or R6G samples in free form, compared to the nanogel formulations, with two types of cells *E. coli* and *Lactobacilli*.

When measuring the fluorescence of the R6G-SPD-NBD conjugate, the fluorescence signals at 490 and 550 nm correspond mainly to NBD and rhodamine, respectively (NBD > R6G and NBD < R6G). Therefore, we can independently monitor the change in the signal from NBD and rhodamine (as part of the conjugate) as the fluorophore adsorbs and penetrate into the cells. To characterize the efficiency of the interaction of fluorophores with cells, we have obtained kinetic curves for the changes in fluorescence of NBD and rhodamine and determined the slope coefficients of the kinetic curve tg(I_550_) and tg(I_490_). The difference between tg(I_550_) and tg(I_490_) is analytically significant, which characterizes simultaneously the quenching of the NBD donor and excitation of the R6G acceptor, such that a quantitative measure of FRET efficiency is realized.

Apparently, the interaction of R6G-spd-NBD, in both its free and nanogel form, is characterized by positive derivatives (tangents), which corresponds to the ignition of R6G fluorescence upon conjugate penetration into cells (Figure 5).

In order to elucidate the mechanisms underlying the observed alteration in fluorescence upon interaction between R6G-spd-NBD fluorescent probes with cells, a series of experiments were conducted using Petri dishes containing *E. coli* bacteria. The fluorescence intensity was visualized after the probes penetrated the cells (Figure 5b).

The results revealed a significant increase in fluorescence intensity upon penetration for the conjugated probe, whereas the fluorescence for the unconjugated R6G probe remained practically unchanged. This finding suggests that the mechanism responsible for the observed effect is primarily due to FRET, which can be visualized through the fluorescent channel within the Petri dish containing the *E. coli* cultures (Figure 5b).

#### 2.3.3. pH Sensitivity of R6G-spd-NBD in the Context of Its Interaction with Bacterial Cells

Only in the case of chitosan nanoparticles are the negative values of the tg are observed for both fluorophores. Here, we observe two phenomena: (1) the ignition of fluorophores as they enter bacterial cells and (2) the transition of the probe from an alkaline (chitosan cation) to an acidic microenvironment on the cell surface due to the pH sensitivity of the fluorophore (Figure 2a). Nonetheless, as a consequence, the concentration of rhodamine and its conjugate diminishes, which implies that in this case the pH-sensitivity factor is prevailing, with FRET playing a secondary role.

The analytically important parameter is the selectivity index: the ratio between the tg(I_550_)–tg(I_490_) values for *E. coli* and *lactobacilli*, as shown in Figure 5a on the right panel. If the selectivity index deviates from 1 to a greater or lesser extent, it indicates the capacity for discriminating between *E. coli* and *lactobacilli* using these formulations with varying degrees of selectivity.

### 2.4. Antibacterial Effect of R6G-spd-NBD Nanogel Formulations and Correlation with Intracellular Permeability

In order to elucidate the correlation between the cell permeability and the antibacterial efficacy, we embarked on a series of experiments. The ability of fluorophores to penetrate bacterial cells was determined by measuring the fluorescence intensity of the solution before and after exposure to cells (Table 2). For R6G, the highest penetration is observed for the free form in *E. coli* cells (the ratio of extracellular to intracellular concentration (C_in_/C_out_) is 3.2), while the lowest penetration occurs for R6G in chitosan nanoparticles form in *Lactobacilli* cells with a C_in_/C_out_ ratio of 1.4. In the latter case, there is a selective effect against *E. coli* compared to *Lactobacilli.*

In the chitosan-based nanogel, R6G exhibits a remarkable enhancement in antibacterial activity, surpassing up to 20% in terms of cellular viability against model pathogenic bacterial strains (*E. coli*), as compared to non-pathogenic ones (*Lactobacilli*) (see Table 1). This finding is promising, but it still remains insufficient for its potential application in therapy.

The conjugate R6G-spd-NBD has demonstrated remarkable selectivity, with targeted penetration into the bacterial cells of *E. coli* versus *Lactobacilli*, with a difference in penetration of more than two times, resulting in up to 40% enhancement in the effectivity (in terms of cell viability) against pathogenic bacteria compared to normal ones (see Table 2).

This effect is achieved through a more precise targeting of the R6G-spd-NBD conjugate compared to free rhodamine, as well as by employing the nanogel form, which selectively binds to cells, enhancing drug availability. Simultaneously, the nanoparticles themselves do not adversely affect cell viability.

These findings allow us to propose the use of nanogel formulations of R6G-spd-NBD as a potential theranostic agent (for the diagnosis and treatment of bacterial infections).

### 2.5. Confocal Laser Scanning Microscopy (CLSM) for Visualization of Bacterial Cell Staining

The process of targeting bacteria (selective imaging) is influenced by two key factors:

(i) The fluorescent probe exhibits pH sensitivity (due to changes in the spermidine protonation and R6G structure), resulting much more intensive fluorescence emission in mildly acidic microenvironments. Additionally, an enhancement in fluorescence resonance energy transfer (FRET) upon fluorophore internalization in cells is observed (crowd effect); and (ii) nanogel particles based on chitosan or heparin exhibit distinct modes of interaction with the membranes of various cell types, with the greatest permeability observed for pathogenic bacterial species. However, nanoparticles exhibit a significantly lower level of interaction with standard cells, specifically HEK293, as illustrated in Figure S5.

**Figure 6:**
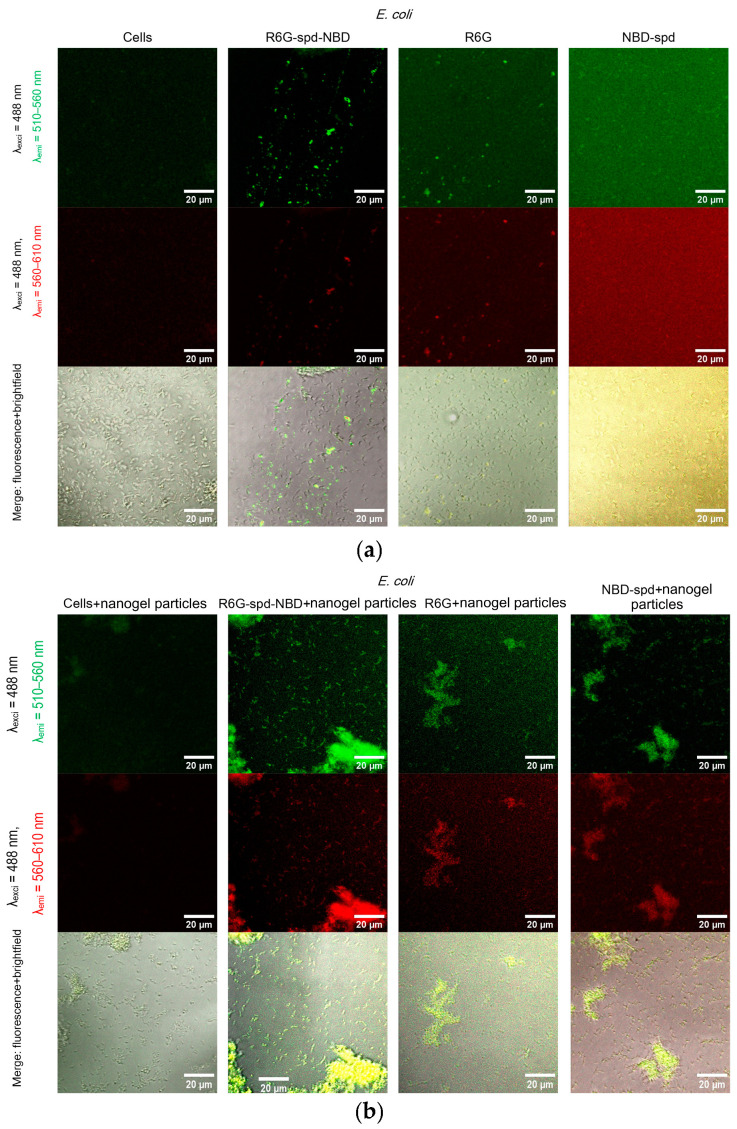

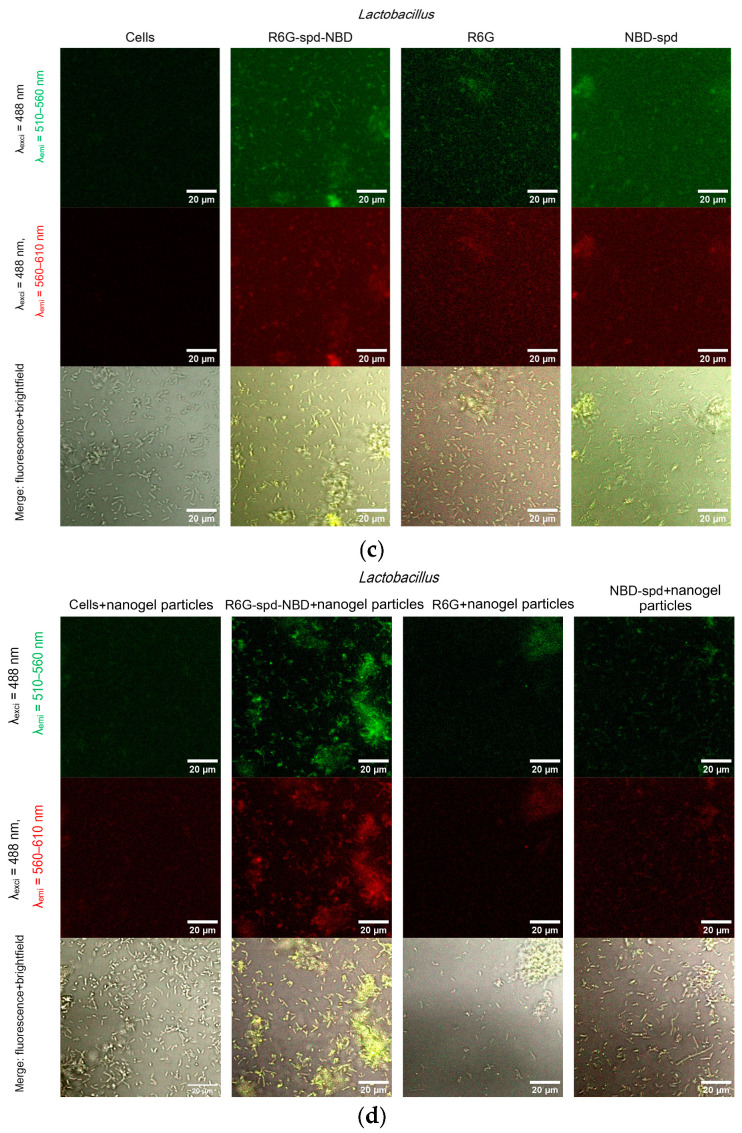
Confocal laser scanning microscopy images of (**a**,**b**) *E. coli* cells and (**c**,**d**) *Lactobacilli* cells, labelled with NBD-spd, R6G, or R6G-spd-NBD (1 µg/mL for all markers) in free or nanogel form. λ_exci_ = 488 nm, λ_emi_ = 510–560 nm (green channel), λ_emi_ = 560–610 nm (red channel). The scale bar is 20 µm. C_mic_ = 0.1 mg/mL.

Figure 6 shows confocal laser scanning microscopy images of *E. coli* cells and *Lactobacilli* cells, labeled with NBD-spd, R6G, or R6G-spd-NBD (1 µg/mL for all markers) in free form or loaded into Chit-LA polymeric nanogels.

In the case of *E. coli* and free fluorophores stain cells (all samples), however, the highest cell/background selectivity is achieved for R6G-spd-NBD. In the case of *E. coli* and fluorophores in a nanogel formulation, an increase in selectivity is observed for all fluorophores in the cell/background ratio, while the R6G-spd-NBD formulation remains the leader.

In the case of *Lactobacilli*, free fluorophores stain cells (but about 20–30% weaker in terms of fluorescence intensity than for *E. coli*); however, the highest cell/background selectivity is achieved for R6G. In the case of *Lactobacilli* and fluorophores in nanogel particles, an increase in the selectivity of R6G-spd-NBD staining in the aspect of cells/background is observed, while NBD-spd and R6G practically do not stain *lactobacilli* and can be used to differentiate *E. coli* against *lactobacilli*. Conversely, R6G-spd-NBD in the composition of chitosan nanoparticles stains *lactobacilli* cells more than 60% more intensively. Thus, we have selected a whole set of diagnostic tools for the visualization of bacteria.

At the same time, R6G-spd-NBD, in its nanogel form, penetrates minimally into normal eukaryotic HEK293T cells (Figure S5). This indicates a very high level of efficiency, with a selectivity index of approximately 10–12 (compared to the free forms, which have an index of approximately 5–7). This difference is due to the combined influence of the pH sensitivity of the dye and the selective permeability of nanoparticles in cells.

### 2.6. The Use of Fluorescence Microscopy for the Visualization of Model Latent Bacterial Infections Localized in Macrophages

An important biomedical challenge is the diagnosis and treatment, or ideally both simultaneously, of latent bacterial infections that occur within macrophages. An example of a disease that requires a new approach, the use of theranostics, in relation to such a dangerous intracellular infection is brucellosis. Brucellosis is an infectious disease characterized by a variety of pathological changes in multiple organs and a propensity for chronicity.

**Figure 7:**
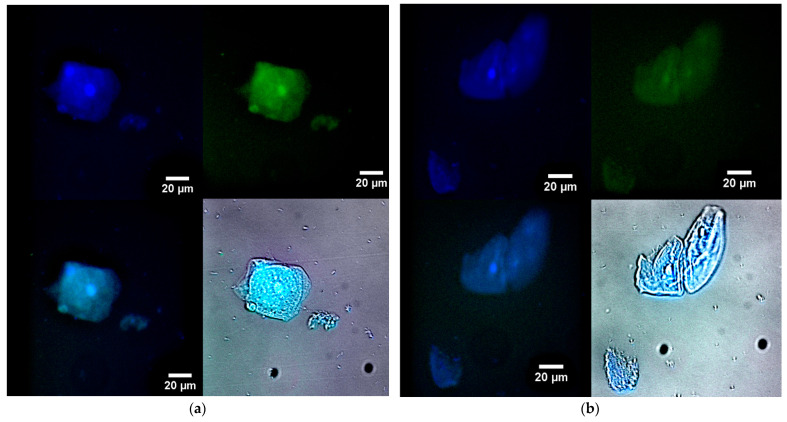
Fluorescence microscopy images of macrophage (large) and bacterial (small) cells from human bronchoalveolar lavage (BAL) labelled with (**a**) R6G-spd-NBD or (**b**) R6G (1 µg/mL for all fluorophores) in nanogel form. Blue channel: λ_exci, max_ = 360 nm, λ_emi_ = 425–700 nm. Green channel: λ_exci, max_ = 475 nm, λ_emi_ = 515–700 nm. Additionally, the blue and green overlay channels and the fluorescence overlay channel on brightfield are shown. The scale bar is 20 µm. C_mic_ = 0.1 mg/mL.

To explore the feasibility of designing therapeutic agents capable of targeting bacterial inclusions within macrophages, we have studied macrophages from human bronchoalveolar lavage (BAL) samples that have phagocytosed bacterial cells as model systems. Figure 7 illustrates fluorescence microscopy images of macrophages (large) and bacteria (small) from human BAL, labeled with either R6G-spd-NBD (Figure 7a) or R6G (1 μg/mL, all fluorophore concentrations) in a nanogel form (Figure 7b). In the blue channel, macrophages’ nuclei and lysosomes, which apparently contain bacteria, are visible. In the green channel, when using R6G-spd-NBD, small dots corresponding to intracellular bacteria can also be seen, in addition to macrophages’ nuclei (a bright spot in the center). The ignition of the nanogel-conjugate takes place in a mildly acidic environment, such as in lysosomes, where the pH ranges from 5.5 to 6.5. The data on the staining parameters of cellular structures within macrophages are presented in Table 3.

Rhodamine also clearly visualizes cellular structures when observed in the blue channel, although the contrast in the green channel is lower (about 3 times weaker than for the conjugate). The effect of the R6G-spd-NBD conjugate can be explained by its pH sensitivity and the enhancement of FRET during the penetration of the fluorophores into the cells. Therefore, the nanogel formulation R6G-spd-NBD has the potential to be used for both the diagnosis of simple bacterial infections and the detection of complex, latent macrophage-associated pathogens such as brucellosis, leishmaniasis, and tuberculosis.

As a control group, we employed CD206+ macrophages derived from non-infected human monocytes (without incubation with *E. coli* cells) (Figure S6). In contrast to the infected activated macrophages obtained from patients, which, following phagocytosis, exhibit a substantial size, ranging from 20 to 30 µm, and loose structure, the model lines of macrophages demonstrate a denser structure, with the average size falling within the range of 10 to 15 µm.

We have demonstrated a remarkable increase in antibacterial activity, reaching up to 45% in terms of cellular survival. These probes exhibit a selective targeting of bacterial infections, which can be visualized, opening up opportunities for phototherapy involving the generation of reactive oxygen species.

The proposed systems represent a theranostic agent, offering a new approach to addressing challenging infectious diseases. These systems rely on the development of technologies for the targeted delivery of antibiotics specifically to macrophages containing dormant bacteria. This approach enables the combination of antibiotic treatment with fluorescence monitoring, allowing for a precise evaluation of the therapeutic process.

## 3. Conclusions

The therapeutic potential of theranostic approaches holds promise for enhancing the management of chronic inflammatory conditions associated with macrophage function, such as leishmaniasis, brucellosis, tuberculosis, and atypical pneumonia caused by Chlamydia pneumoniae. Infections within macrophages, particularly those caused by pathogens like *Brucella*, present significant challenges due to their capacity for persistent latency. We have developed a groundbreaking nanogel formulation comprising rhodamine 6G (R6G) conjugated with 4-nitro-2,1,3-benzoxadiazole (NBD) and linked by a spacer molecule spermidine (spd). This nanogel represents a highly promising theranostic agent, combining the functionalities of pH responsiveness and fluorescence resonance energy transfer (FRET). The R6G-spd-NBD nanogel probe exhibits remarkable sensitivity to changes in the acidity levels of the cellular environment. The nanogel particles, formed by amphiphilic polymers loaded with probes, exhibit a compact and spheroidal shape, ranging from 70 to 100 nanometers in diameter. R6G-spd-NBD-loaded nanogel particles exhibit selective staining properties toward *E. coli* and *lactobacilli*, enabling the visualization of macrophages containing bacteria through confocal laser scanning microscopy.

Microbiological studies have shown that the R6G-spd-NBD nanogel exhibits increased toxicity compared to its free form and control samples of rhodamine 6G. Human bronchoalveolar lavage (BAL) macrophages were used to investigate the potential of developing theranostic nanogel particles for visualizing macrophages and bacteria in BAL samples. The formulation of R6G-spd-NBD nanogels has been shown to be effective for diagnosing both simple bacterial infections and more complex latent pathogens. Consequently, these fluorescent probes constitute promising theranostic agents that can be employed in the diagnosis and treatment of complex infections involving macrophages.

## 4. Materials and Methods

### 4.1. Reagents

NBD-Cl (4-chloro-7-nitro-1,2,3-benzaxadiazole) was purchased from Thermofisher Scientific (Carlsbad, CA, USA). Rhodamine 6G (R6G), spermidine (spd), 4-hydroxybenzaldehyde, chitosan oligosaccharide lactate 5 kDa (Chit5), and lipoic acid (LA) were obtained from Sigma-Aldrich (St. Louis, MI, USA). Para-toluenesulfonic acid (TsOH), salts for the preparation of buffer solutions, NaOH, and HCl were produced by Reachim (Moscow, Russia).

### 4.2. Synthesis of R6G-spd-NBD Fluorophore

The synthesis of the pH-responsive fluorophore R6G-spd-NBD was accomplished in four stages, as previously outlined [54]. Briefly, in 1st stage, the samples of spd and 4-hydroxybenzaldehyde were reacted with the formation of spd-N=CH-C_6_H_4_-OH. Then, in the 2nd stage, NBD-Cl was modified with spd-N=CH-C_6_H_4_-OH; as a result, yellow–orange flakes of NBD-spd-N=CH-C_6_H_4_-OH were isolated. In the 3rd stage, 3 NBD-spd-N=CH-C_6_H_4_-OH was deprotected with the formation of the NBD-spd product. Finally, NBD-spd was reacted with R6G. The product R6G-spd-NBD, with a mass of 55 mg and a quantity of 0.074 mmol, was obtained with a yield of 76% after undergoing four stages of synthesis, resulting in a final yield of 46%. The drug is moderately soluble in PBS up to a concentration of 0.3 mg/mL, which is equivalent to 0.4 mM.

The degree of modification of the NH_2_ groups in spermidine (spd) was determined through spectrophotometric titrations before and after the modification process using 2,4,6-trinitrobenzene sulfonic acid (TNBS) and a 0.02M sodium borate buffer solution with a pH of 9.2. The number of NH_2_ groups that were titrated in this manner was as follows:

1. Spermidine: two groups per molecule.

2. spd-N=CH-C_6_H_4_-OH: one group per molecule.

3. NH_2_- per NBD-SPD-N=CH-C_6_H_4_-OH: none.

4. NBD-spd: one group per molecule.

5. R6G-spd-NBD: one group per molecule.

### 4.3. Synthesis of Chit5-LA and Hep-LA Amphiphilic Conjugates and Preparation of Polysaccharide-Based Nanogel Theranostic Formulations

#### 4.3.1. Synthesis of Chit5-LA and Hep-LA Amphiphilic Conjugates

The synthesis of lipoic acid (LA)-modified 5 kDa chitosan (Chit-LA) and lipoic acid (LA)-modified 12–14 kDa heparin (Hep-LA) was carried out as described by us earlier [39].

Briefly, for **Chit5-OA and Chit5-LA**, a solution of 30 mg of oleic acid (OA) and lipoic acid (LA) in 5 mL of CH_3_CN/PBS (a 4:1 volume ratio, pH 7.4) was prepared. A 2.5-fold excess of EDC and a 1.3-fold excess of NHS (N-hydroxysuccinimide) dissolved in DMF were added to the mixtures. The samples were allowed to react for 20 min at 50 °C. Subsequently, 90 mg of pre-dissolved Chit5 (5 mg/mL in phosphate buffer with pH 6.5) was added to each reaction mixture. The mixtures were then incubated at 50 °C for 6 h. The reaction products were purified using a centrifuge filter with a cut-off size of 3 kDa and a centrifugation speed of 10,000× g for 3 min, followed by dialysis against water for 12 h with a cut-off range of 6–8 kDa.

The degree of modification of Chit5 was determined using a spectrophotometric titration method with 2,4,6-trinitrobenzene sulfonic acid in a sodium-borate buffer with a pH of 9.2.

**Hep-OA**: Hep-OA is a specific term that requires further context to be fully understood. A solution of 125 mg of heparin (Hep) in 10 mL of PBS was prepared. To this solution, a 2.5-fold molar excess of NHS and a 1.3-fold excess EDC relative to the amount of oleylamine were added in dimethylformamide (DMF). The mixture was allowed to react for 20 min at 50 °C. Subsequently, pre-dissolved oleylamine, weighing 40 mg and dissolved in a mixture of CH_3_CN and PBS (5 mL, volume ratio 4:1) adjusted to a pH of 7.4, was added to the reaction mixture. The mixture was then incubated for 6 h at 50 °C.

**Hep-LA**: In order to synthesize a conjugate of heparin and lipoic acid, known as Hep-LA, we employed a carbodiimide protocol. Initially, we prepared heparin modified with spermine, achieving a grafting degree of 20%. Then, we attached lipoic acid residues to the Hep-sp molecules via the amino groups of the spermine moiety.

The reaction mixtures of Hep samples were subsequently purified using centrifugal filters (cut-off 10 kDa, 10,000× *g*, three cycles of 10 min each). The purified products were dialyzed against deionized water for 12 h, using a dialysis membrane with a cutoff range between 12 and 14 kDa. Finally, all samples were lyophilized at –60 °C using a freeze dryer from Edwards (BOC Edwards, Crawley, UK).

#### 4.3.2. Preparation of Polysaccharide-Based Nanogel Theranostic Formulations

Chit-LA or Hep-LA polymer (2 mL, 1 mg/mL) was mixed with R6G, NBD-spd, or theranostic sensor R6G-spd-NBD (2 mL, 0.1 mg/mL) in PBS (0.01 M, pH of 7.4), and the mixtures were then incubated at 37 °C for 1 h. The nanogel compositions were prepared via ultrasonic treatment of samples (22 kHz) for 10–15 min with constant cooling in an ultrasonic device (Cole-Parmer Instrument, Vernon Hills, IL, USA). Polysaccharide-based samples were 5-fold extruded (200 nm membrane, Avanti Polar Lipids, Alabaster, AL, USA). Subsequently, the free fluorophores were separated by means of dialysis against PBS, with a cut-off molecular weight ranging from 6 to 8 kDa. The loading level was then quantified through absorbance measurements at wavelengths of 490 and 515 nm. The stability and release curves of rhodamine 6G are presented in Figure S7.

### 4.4. Characterization of Nanogel Particles

#### 4.4.1. Fourier Infrared Spectroscopy

The FTIR spectra of the samples in suspension were recorded using a Bruker Tensor 27 spectrometer (Bruker, Ettlingen, Germany) and FTIR microscope MICRAN-3 (Simex, Novosibirsk, Russia) equipped with a liquid N_2_-cooled MCT (cadmium–mercury telluride) detector, as previously outlined [54]. The spectra were recorded in the mode of Attenuated total reflection (ATR).

#### 4.4.2. NMR Spectroscopy

The ^1^H NMR and ^13^C NMR spectra were registered on a Bruker DRX-500 device [working frequencies of 500.13 MHz (^1^H) and 125.76 MHz (^13^C)]. Chemical shifts were expressed in parts per million (ppm) and assigned to the appropriate NMR solvent peaks.

#### 4.4.3. Fluorescence Spectroscopy

Fluorescence emission and excitation were recorded on Varian Cary Eclipse fluorescence spectrophotometer (Agilent Technologies, Santa Clara, CA, USA) at T = 22 and 37 °C. λ_exci_ = 460 or 515 nm. λ_emi_ = 550 nm.

#### 4.4.4. Circular Dichroism Spectroscopy

Circular dichroism (CD) spectra were registered on Jasco J-815 CD Spectrometer (JASCO, Tokyo, Japan) and were used to calculate the chitosan deacetylation degree, which amounted to (95 ± 2%).

#### 4.4.5. Atomic Force Microscopy

Nanoparticles (0.1 mg/mL per polymers in nano pure water) were treated with ultrasound for 15 min before examination. Then, about 5 µL of a nanoparticle sample was applied to the freshly chopped mica, and the droplet was allowed to drain freely at an angle and then washed gently with water. Atomic force microscopy (AFM microscope NTEGRA II, NT-MDT Spectrum Instruments, Moscow, Russia) was used to study the nanogel particles’ formation, topology, and size.

#### 4.4.6. Dynamic Light Scattering

Dynamic Light Scattering (DLS) was used to determine the nanogel particle sizes and ζ-potentials using a Zetasizer Nano S “Malvern” (Worcestershire, UK). Chitosan-lipoic acid nanoparticles (Chit-LA) have a positive charge (ζ = +8 mV), in the contrary, heparin-lipoic acid particles (Hep-LA) have a negative charge (ζ = –12 mV). Non-loaded particles are approximately 210 ± 40 nm in size (hydrodynamic diameter). Chit-LA nanogel particles containing R6G-spd-NBD are approximately 100 ± 20 nm in size. Hep-LA nanogel particles, empty and containing R6G-spd-NBD, are approximately 310 ± 50 nm and 250 ± 70 nm in size, correspondingly.

### 4.5. Microbiology Experiments

#### 4.5.1. Bacterial Cells Cultivation

The strains used in this study were *Escherichia coli* (ATCC 25922) from the National Resource Center Russian collection of industrial microorganisms, SIC “Kurchatov Institute”, and *Lactobacillus* (commercially available pharmaceutical Lactobacillus Liquid Concentrate). The cultures were cultivated for 18–20 h at 37 °C to a CFU/mL ≈ 2×10^7^ (determined by measuring A_600_ and by seeding on petri dishes) in the liquid nutrient medium Luria–Bertani (pH 7.2) with stirring at 120 rpm.

#### 4.5.2. Antibacterial Studies

The cell suspensions (10^7^ CFU/mL) were incubated with the R6G, NBD-spd, or R6G-spd-NBD-containing samples (free and nanogel form). The specimens were incubated at 37 °C for 24 h. After incubation, the number of surviving cells was determined by A700 and controlled by quantitative seeding on Petri dishes. The dishes were placed in the incubator at 37 °C for 24 h. Then, the number of colonies (CFU) was counted.

#### 4.5.3. Visualization of Petri Dishes with Bacteria Incubated with Fluorophores

Fluorescent image of a Petri dish with *E. coli* cells (10^7^ CFU/0.5 mL placed onto 20 mL of agar medium) when exposed to R6G or R6G-spd-NBD and the nanogel form for 12 h at 37 °C were obtained with a UVP BioSpectrum imaging system (BioSpectrum; UVP, Upland, CA, USA). The fluorophores are placed in wells with a diameter of 9 mm. The concentration of fluorophores was 0.1 mg/mL, and the concentration of amphiphilic polymers was 0.5 mg/mL. λ_exci, max_ = 480 nm. λ_emi_ = 515–570 nm.

### 4.6. Confocal Laser Scanning Microscopy (CLSM) and Fluorescence Microscopy

#### 4.6.1. CLSM Visualization of Bacterial Cell Staining Using a Theranostic Preparation

Bacterial cells (5 × 10^6^ cells/mL) were incubated with 1 µM of NBD-spd, R6G, or R6G-spd-NBD in a free or nanogel (0.1 mg/mL Chit-LA or Hep-LA) form, followed by double washing (5 min, 8000× *g*), and transferred to the wells of a 96-well plate. The cells were fixed with parafarm and filled with 70 µL of 50% glycerin-PBS.

CLSM images were obtained using the confocal laser scanning microscope Olympus FluoView FV1000 equipped with both a spectral version scan unit with emission detectors and a transmitted light detector, as previously outlined [54]. The dry objective lens Olympus UPLSAPO 40X NA 0.90 was used for the measurements. Fluorescence was collected using emission windows set at 510–560 nm (green channel) and 560–610 nm (red channel).

#### 4.6.2. Fluorescence Microscopy for Visualization of Model Latent Bacterial Infections Localized in Macrophages

Macrophages with CD206 expression, both with and without absorbed bacteria, were captured on a polymer chip coated with mannan [29,60,61]. The macrophages were then incubated with theranostics agents for 3 h. Following this, the macrophages were washed to remove any free fluorophores and transferred to the wells of 8-well plate, where they were fixed with paraformaldehyde and analyzed using fluorescence microscopy.

Fluorescence images were obtained using a IX81 motorized inverted microscope (Olympus, Tokyo, Japan) [62]. The use of a fluorescence microscope instead of a confocal laser scanning microscope was due to the fact that ultraviolet light was required to excite fluorescence (Blue channel: λ_exci, max_ = 360 nm, λ_emi_ = 425–700 nm. Green channel: λ_exci, max_ = 475 nm, λ_emi_ = 515–700 nm). Additionally, the blue and green overlay channels and the fluorescence overlay channel on brightfield are shown. The scale bar is 20 µm.

## Figures and Tables

**Table 1:** The main physico-chemical parameters of the nanogel particles based on grafted chitosan and heparin.

Designation *	Polymer Modification Degree per Glycoside Unit, %	The Average Molecular Mass of One Structural Unit, kDa	Hydrodynamic Diameter, nm	ζ-Potential, mV	Aggregative Stability
Chit5-LA	25 ± 3	6.4 ± 0.5	210 ± 40	+8 ± 1	>36 h
Hep-LA	10 ± 2	14 ± 2	310 ± 50	–12 ± 1.5	>48 h

* Chit5—chitosan 5 kDa, Hep—heparin 12–14 kDa, LA—lipoic acid residue.

**Table 2:** Antibacterial effect and cellular permeability of R6G-spd-NBD nanogel formulations on *E. coli* and *Lactobacillus* cells after 8 h of incubation. LB medium at pH 7.2, 37 °C, cell concentration 10⁻⁷ CFU/mL, fluorophore concentration 0.05 mg/mL, amphiphilic polymer concentration 0.25 mg/mL. C_in_—intracellular concentration of fluorophore, C—extracellular concentration of fluorophore.

Sample *	Cell Viability, %		C_in_/C_out_	
	*E. coli*	*Lactobacilli*	*E. coli*	*Lactobacilli*
**R6G**	84 ± 5	87 ± 2	3.2 ± 0.3	2.2 ± 0.1
**R6G in Chit-LA in nanogel**	67 ± 4	85 ± 2	2.3 ± 0.1	1.4 ± 0.1
**R6G in Hep-LA in nanogel**	88 ± 7	81 ± 4	2.5 ± 0.2	1.7 ± 0.2
**R6G-spd-NBD**	70 ± 3	92 ± 1	2.1 ± 0.2	1.4 ± 0.1
**R6G-spd-NBD in Chit-LA in nanogel**	45 ± 2	89 ± 4	3.6 ± 0.4	1.7 ± 0.3
**R6G-spd-NBD in Hep-LA in nanogel**	62 ± 6	93 ± 3	2.8 ± 0.2	1.3 ± 0.1
**Chit-LA in nanogel**	97 ± 1	>99	Polymers mostly adsorbed onto cell surface	
**Hep-LA in nanogel**	>99	>99		

* The concentration of rhodamine was taken near to a semi-inhibitory level, just above the midpoint of the range, in order to allow for the enhancement of antibacterial activity through the use of nanogel formulations and conjugation.

**Table 3:** Quantitative parameters of staining of cellular structures in macrophage cells: fluorescence intensity on a scale of 0–255. T = 37 °C.

Probe	Blue Channel				Green Channel			
	Background	Nucleus	Lysosomes	Cytoplasm	Background	Nucleus	Lysosomes	Cytoplasm
**R6G-spd-NBD**	14	253	250 and 134	130	2	164	105 and 108	91
**R6G**	22	198	130	105	16	84	62	54

## Data Availability

The data presented in this study are available in the main text and in Supplementary Materials.

## Supplementary Materials

The following supporting information can be downloaded at https://www.mdpi.com/article/10.3390/gels10090567/s1, Figure S1. (a) Synthesis scheme of the pH-sensitive fluorophore R6G-spd-NBD. The products are a mixture of isomers (because spermidine is not symmetrical). (b) Chemical formula of Hep-LA conjugate. Figure S2. FTIR spectra of initial compounds, intermediate substances, and product of synthesis of pH-sensitive fluorophore R6G-spd-NBD: (a) stage 1, (b) stage 2, (c) stage 3, (d) stage 4. Aqueous solutions. T = 22 °C. Figure S3. ^1^H NMR spectra of NBD-spd. T = 22 °C. DMSO-d_6_, 500 MHz. Figure S4. Fluorescence excitation spectra of rhodamine 6G (R6G), NBD and R6G-spd-NBD at a fixed emission wavelength of 550 nm. T = 37 °C. Figure S5. Confocal laser scanning microscopy images of HEK293T normal cells labeled with R6G or R6G-spd-NBD (1 µg/mL for all markers). λexci = 488 nm, λemi = 510–560 nm (green), λemi = 560–800 nm (red). The green and red channels are shown, as well as a brightfield and merge. The scale segment is 60 µm. Chit5-LA nanogel particles. Cmic = 0.1 mg/mL. Figure S6. Confocal laser scanning images of CD206+ macrophages (from human monocytes, non-infected) after 2h-icubation with HPCD-PEI-triMan-FITC. The scale segment is 100 µm (division value is 20 µm); λ_exci_ = 488 nm. Figure S7. Release curves of rhodamine 6G (R6G) from Chit5-LA-based nanoparticles. T = 37 °C. 0.01 M PBS (pH 7.4).
