# Supplementary material for: pH-Sensitive Fluorescent Probe in Nanogel Particles as Theragnostic Agent for Imaging and Elimination of Latent Bacterial Cells Residing Inside Macrophages
Source: Gels. 2024 Aug 30;10(9):567. doi: 10.3390/gels10090567 (PMC11431188; doi:10.3390/gels10090567)

pH-sensitive Fluorescent Probe in Nanogel Particles as Theragnostic Agent for Imaging and Elimination of Latent Bacterial Cells Residing Inside Macrophages

Igor D. Zlotnikov [1], Alexander A. Ezhov [2], Natalya G. Belogurova [1] and Elena V. Kudryashova [1,*]

[1] Faculty of Chemistry, Lomonosov Moscow State University, Leninskie Gory, 1/3, 119991 Moscow, Russia; zlotnikovid@my.msu.ru, nbelog@mail.ru (N.G.B.)
[2] Faculty of Physics, Lomonosov Moscow State University, Leninskie Gory, 1/2, 119991 Moscow, Russia; alexander-ezhov@yandex.ru (A.A.E.)
* Correspondence: helena_koudriachova@hotmail.com (E.V.K.)

Figure S1. (a) Synthesis scheme of the pH-sensitive fluorophore NBD-spd-R6G. The products are a mixture of isomers (because spermidine is not symmetrical). **(b)** Chemical formula of Hep-LA conjugate.

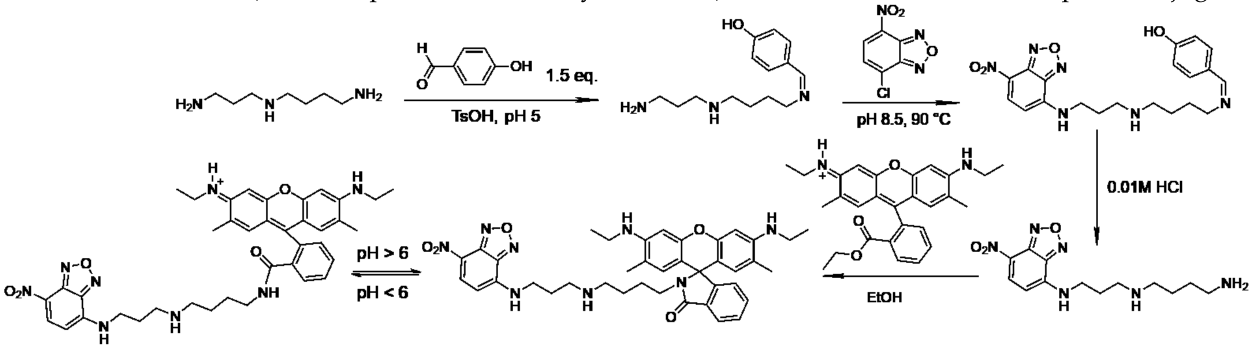

(a)

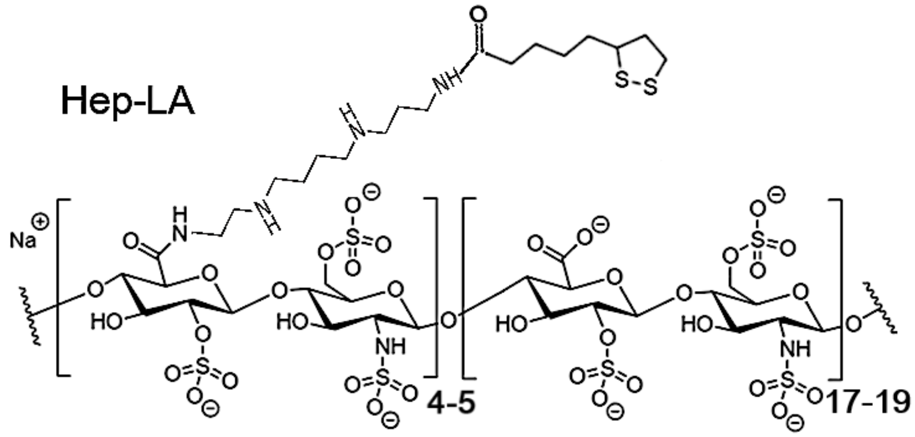

(b)

Figure S2. FTIR spectra of initial compounds, intermediate substances, and product of synthesis of pH-sensitive fluorophore R6G-spd-NBD: (**a**) stage 1, (**b**) stage 2, (**c**) stage 3, (**d**) stage 4. Aqueous solutions. T = 22 °C.

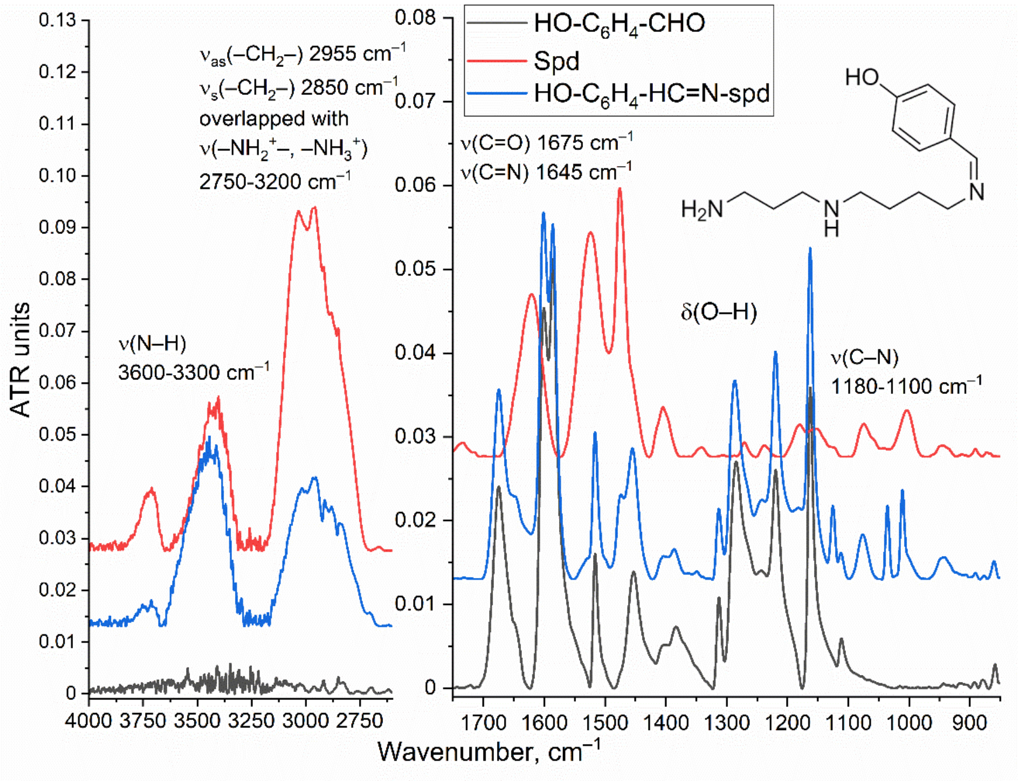

(**a**)

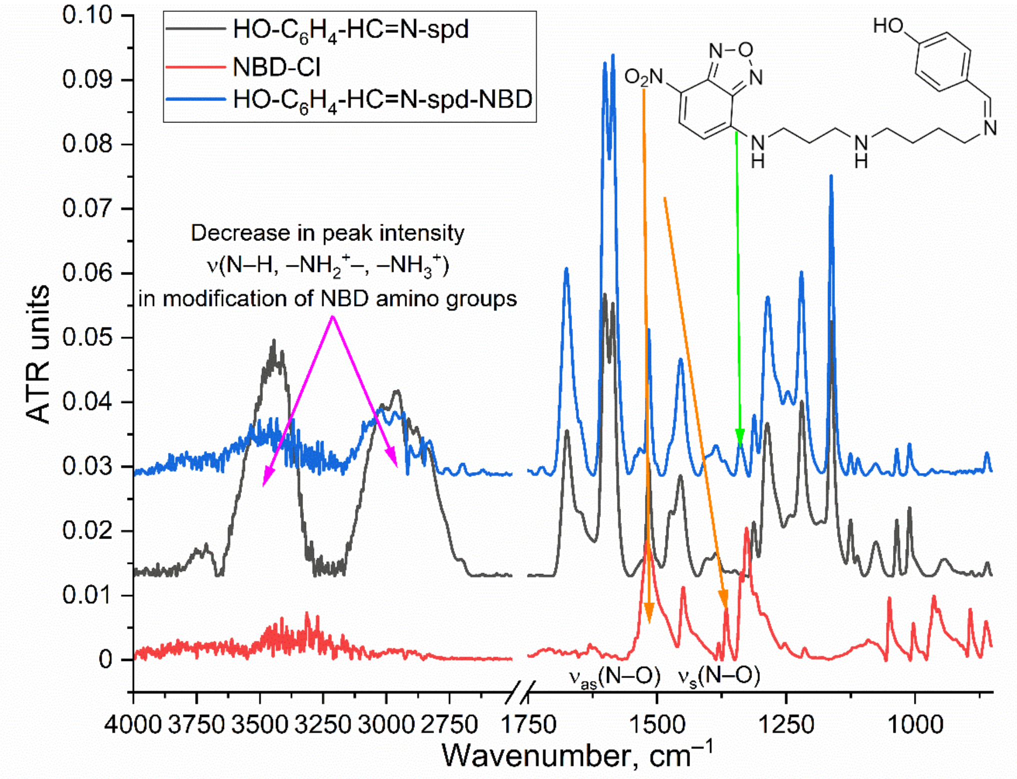

(**b**)

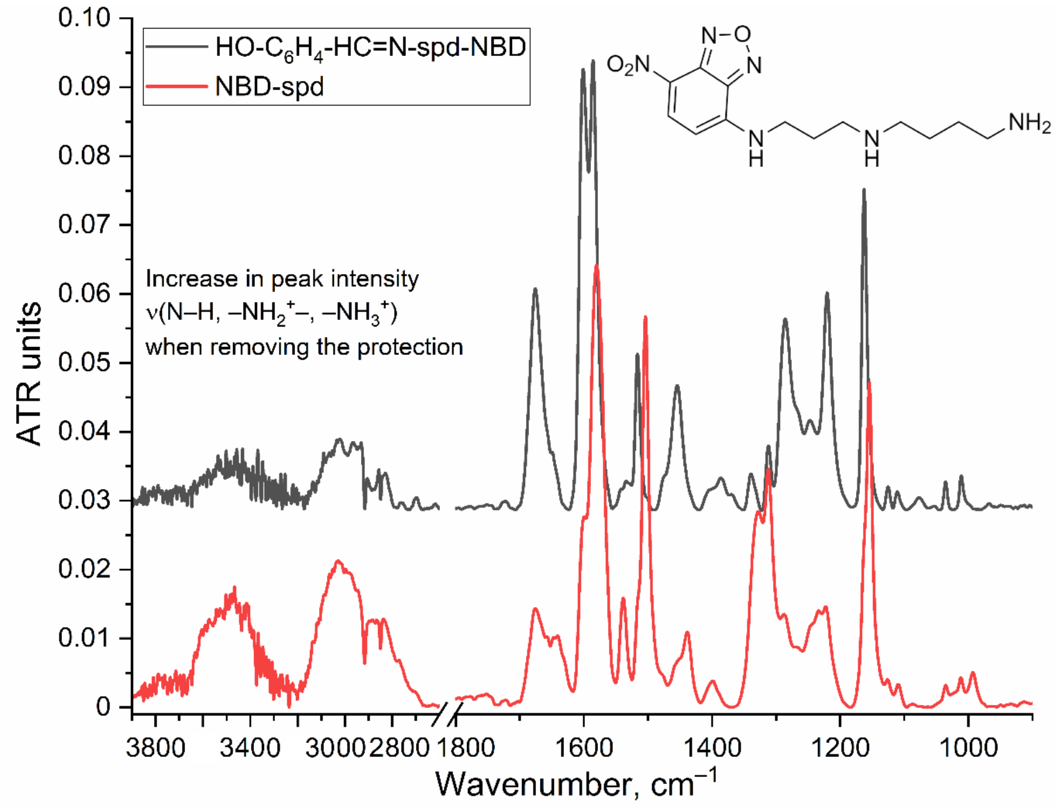

HO-C6H4-HC=N-spd-NBD
NBD-spd
Increase in peak intensity
ν(N–H, –NH2+–, –NH3+)
when removing the protection
ATR units
Wavenumber, cm−1

(c)

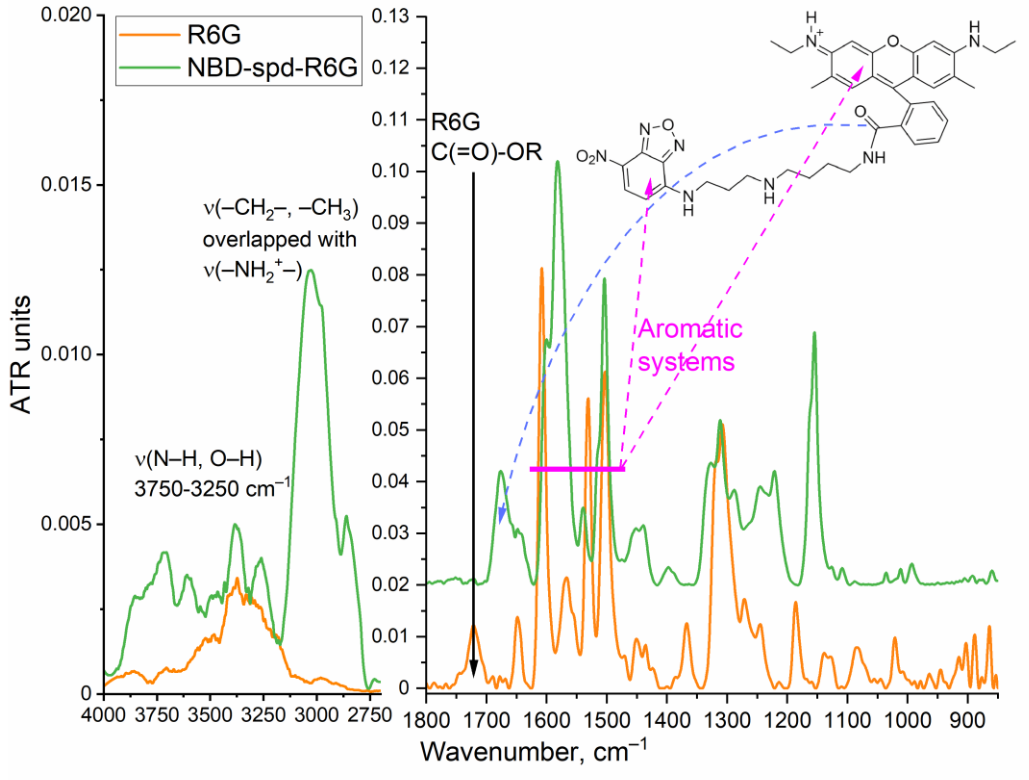

R6G
NBD-spd-R6G
ν(–CH2–, –CH3)
overlapped with
ν(–NH2+–)
ν(N–H, O–H)
3750-3250 cm−1
R6G
C(=O)-OR
Aromatic
systems
ATR units
Wavenumber, cm−1

(d)

Figure S3. ^{1}H NMR spectra of NBD-spd. T = 22 °C. DMSO-d_6, 500 MHz.

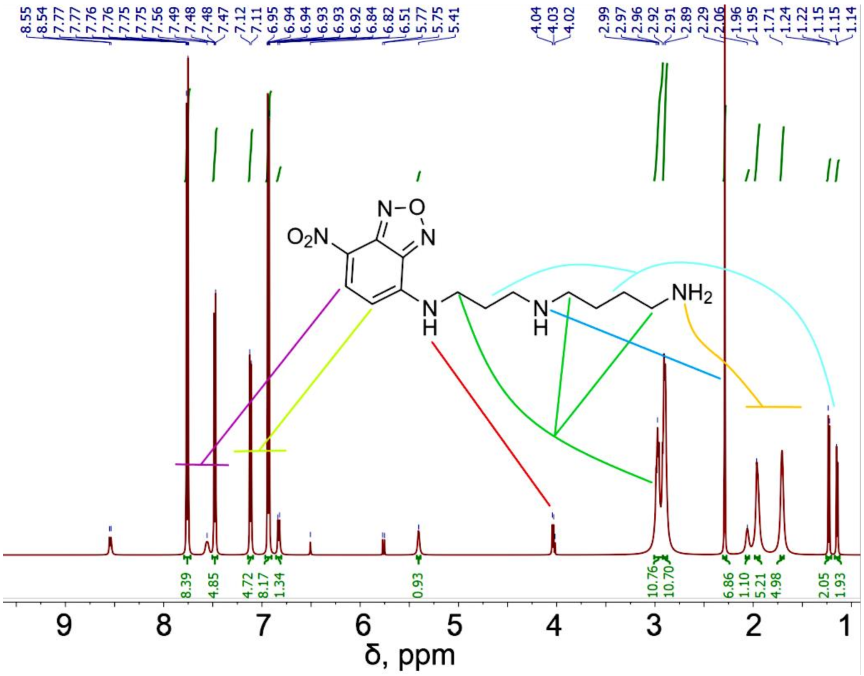

Figure S4. Fluorescence excitation spectra of Rhodamine 6G (R6G), NBD and R6G-spd-NBD at a fixed emission wavelength of 550 nm. T = 37 °C.

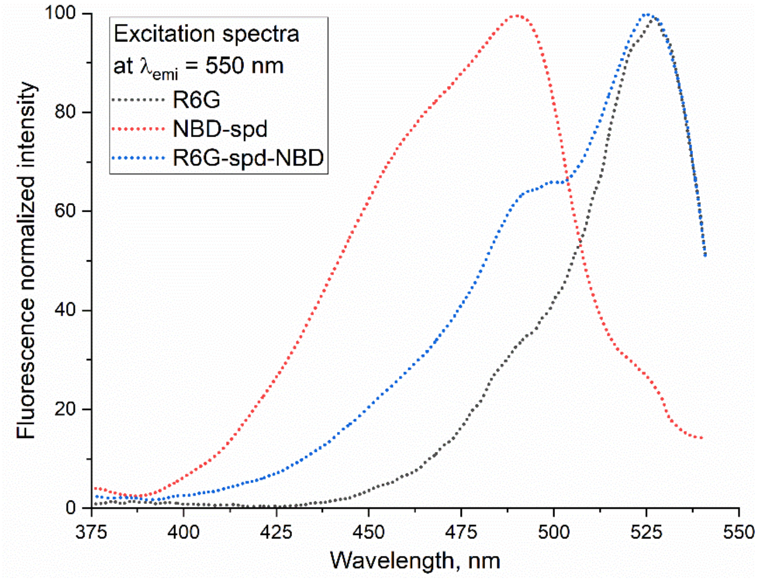

Figure S5. Confocal laser scanning microscopy images of HEK293T normal cells labeled with R6G or R6G-spd-NBD (1 μg/mL for all markers). λexci = 488 nm, λemi = 510–560 nm (green), λemi = 560–800 nm (red). The green and red channels are shown, as well as a brightfield and merge. The scale segment is 60 μm. "Micelles" means Chit5-LA nanogel particles. Cmic = 0.1 mg/mL.

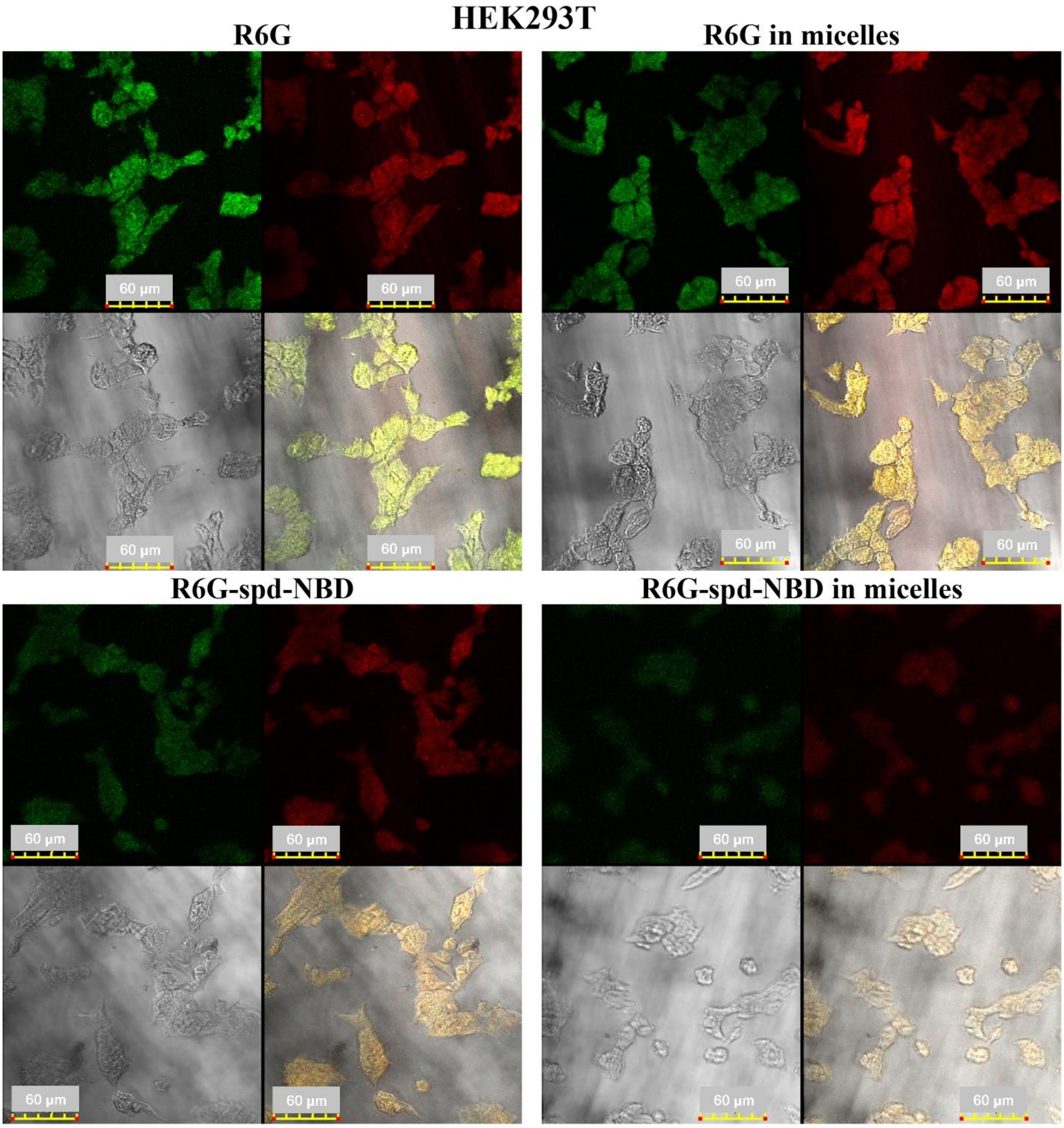

Figure S6. Confocal laser scanning images of CD206+ macrophages (derived from human monocytes, non-infected) after 2h-icubation with HPCD-PEI-triMan-FITC. The scale segment is 100 μm (division value is 20 μm); λ_{exci} = 488 nm.

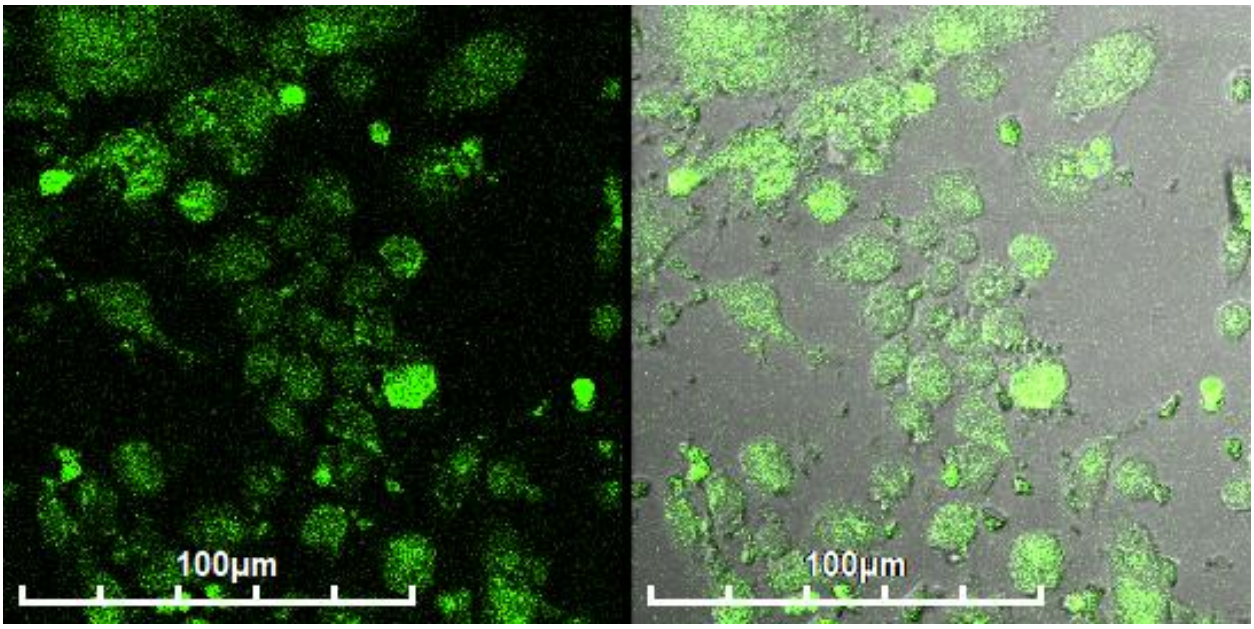

Figure S7. Release curves of rhodamine 6G (R6G) from Chit5-LA-based nanoparticles. T = 37 °C. 0.01 M PBS (pH 7.4).

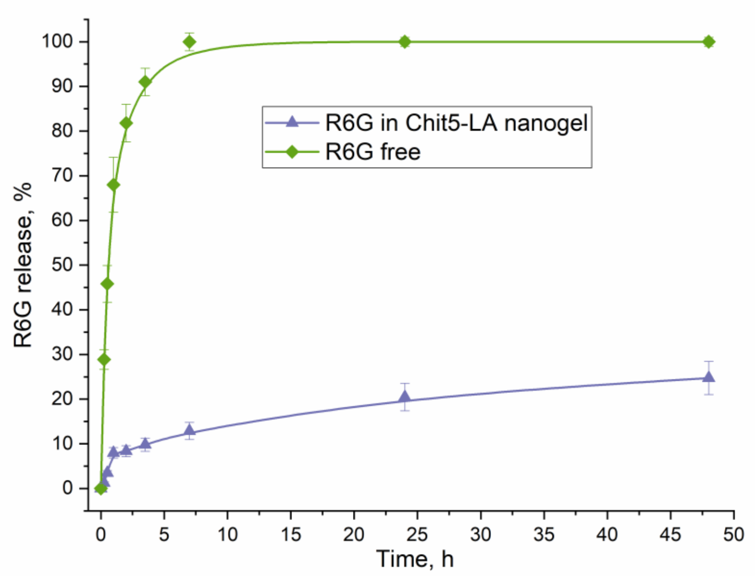

Supplement: Supplementary file 1 [file gels-10-00567-s001.zip › gels-3173853-supplementary.pdf]
